# Video game-based interventions for post-stroke dysphagia: a systematic review and meta-analysis

**DOI:** 10.3389/fneur.2026.1889031

**Published:** 2026-08-28

**Authors:** Qiaoqiao Wang, Jiayang Qu, Lili Lin, Guihua Zhong, Sijia Zhao, Xi Chen, Jun Zhao, Guohao Lin, Xuekang Niu

**Affiliations:** 1 The Third Affiliated Hospital of Zhejiang Chinese Medical University, Hangzhou, China; 2 Zhejiang Chinese Medical University, Hangzhou, China

**Keywords:** biofeedback, meta-analysis, post-stroke dysphagia, rehabilitation, systematic review, video game

## Abstract

**Objective:**

To systematically evaluate the efficacy of video game-based interventions on swallowing function and swallowing-related quality of life in patients with post-stroke dysphagia.

**Methods:**

A systematic search was conducted in PubMed, Web of Science, Cochrane Library, and Embase from inception to July 13th, 2026. Randomized controlled trials (RCTs) evaluating video game-based training for post-stroke dysphagia were included. The primary outcome was the Functional Oral Intake Scale (FOIS). Secondary outcomes included the Standardized Swallowing Assessment (SSA) and swallowing-related quality of life (SWAL-QOL/DHI). The certainty of evidence was assessed using the Grading of Recommendations Assessment, Development and Evaluation (GRADE) framework.

**Results:**

Six randomized controlled trials randomized a total of 309 participants, of whom 300 contributed data to the meta-analysis. Video game-based interventions significantly improved FOIS scores (WMD = 0.78, 95% CI [0.36, 1.21], *P* = 0.0003; low GRADE certainty), though heterogeneity was substantial (*I*^2^ = 74%). Sensitivity analysis identified one outlying study; after its exclusion, heterogeneity was reduced to zero, and the effect remained significant and consistent (moderate certainty). Video game-based training also significantly reduced SSA scores (WMD = −2.14, 95% CI [−3.55, −0.74]; moderate GRADE certainty) and improved swallowing-related quality of life (WMD = −15.07, 95% CI [−21.61, −8.52]; low GRADE certainty due to imprecision).

**Conclusion:**

The evidence suggests that video game-based interventions improve swallowing function and quality of life in post-stroke dysphagia. However, given the limited number of included trials, these findings should be interpreted with caution. Larger, well-designed RCTs are warranted to confirm the clinical utility and to clarify the differential effects of intervention technologies.

**Systematic review registration:**

https://www.crd.york.ac.uk/PROSPERO/view/CRD420261359653, identifier: CRD420261359653.

## 1 Background

Stroke is a leading cause of death and long-term disability worldwide (1), and dysphagia is one of the most common complications after stroke (2). Epidemiological data indicate that the overall prevalence of post-stroke dysphagia is 46.6% (3), which significantly affects rehabilitation prognosis of patients (4). Dysphagia can lead to a series of serious complications, including aspiration, aspiration pneumonia, malnutrition, dehydration, and even death (5, 6). Furthermore, dysphagia prolongs hospital stays, increases healthcare costs, and severely reduces patients' quality of life (7, 8). For stroke patients requiring tracheostomy, the management of dysphagia becomes more complex, and the risk of aspiration is further increased (9).

Currently, rehabilitation interventions for post-stroke dysphagia primarily include compensatory strategies (e.g., postural adjustments, texture-modified diets), swallowing maneuvers (e.g., the Mendelsohn maneuver, effortful swallowing), oral motor exercises, sensory stimulation, neuromuscular electrical stimulation, and repetitive transcranial magnetic stimulation (10–12). However, these approaches have certain limitations. Traditional swallowing rehabilitation exercises are often monotonous and tedious, lacking real-time feedback for patients, which makes it difficult to sustain long-term training motivation and adherence (13, 14). Studies have shown that patient adherence to rehabilitation training directly affects treatment outcomes (15), and monotonous training methods often lead to decreased adherence. Moreover, advanced techniques such as repetitive transcranial magnetic stimulation or transcranial direct current stimulation have high requirements for operator expertise (16); issues such as target localization and parameter settings still lack optimal solutions, which limits their widespread clinical application (17).

Based on the theories of motor learning and neuroplasticity, rehabilitation approaches that provide external feedback have received increasing attention in recent years (18, 19). The application of digital health technologies in swallowing rehabilitation, including mobile health-based home rehabilitation, synchronous telemedicine monitoring, and game-based biofeedback training, offers new possibilities for the treatment of dysphagia (20). Biofeedback techniques can provide patients with real-time audiovisual feedback, enhancing their perception and control of muscle activation and relaxation, thereby improving movement accuracy and coordination (21). Gamified rehabilitation training can significantly increase patients' treatment motivation and engagement, making otherwise monotonous swallowing exercises more interesting and appealing (22). Research suggests that through the cultivation of “flow” experiences and “grit” qualities in game design, patient adherence to swallowing therapy can be effectively improved (23). Functional near-infrared spectroscopy studies have confirmed that game-based rehabilitation training enhances cortical activation and functional connectivity in relevant brain regions (24). Integrating video games with biofeedback may thus provide a novel strategy to enhance swallowing rehabilitation.

Although existing studies have generally shown positive results, the number of studies is limited. The sample sizes of individual studies are generally small, intervention methods show considerable heterogeneity (20), outcome measures are inconsistent, and most studies lack long-term follow-up data. Based on the above research background and evidence gaps, this study aims to conduct a systematic review and meta-analysis to evaluate the efficacy of video game interventions for patients with post-stroke dysphagia, thereby providing evidence-based guidance for clinical practice and informing the development and application of future swallowing rehabilitation technologies.

## 2 Methods

This study was registered with the PROSPERO international prospective register of systematic reviews (registration number: CRD420261359653). This systematic review and meta-analysis was conducted and reported according to the Preferred Reporting Items for Systematic Reviews and Meta-Analyses (PRISMA) guidelines (25).

### 2.1 Search strategy

A systematic search was performed in the following electronic databases: PubMed, Web of Science, Cochrane Library, and Embase. The search period covered from the inception of each database to July 13th, 2026. A combination of controlled vocabulary (MeSH terms) and free-text words was used, with the search strategy adapted to the syntax of each database. The search terms included: (1) terms related to dysphagia: Deglutition Disorders, Dysphagia, Swallowing Disorders, Oropharyngeal Dysphagia, Esophageal Dysphagia; and (2) terms related to video games: Video Games, Computer Games, game-based, computer-based, digital game, serious game. The detailed search strategies for each database are provided in Supplementary material 1. The four databases were selected based on their comprehensive coverage of biomedical literature and clinical trials, which aligns with the clinical and rehabilitation focus of this review. We did not search CINAHL or PEDro, as these databases have a stronger emphasis on nursing and physical therapy literature and were not expected to yield substantial additional relevant studies beyond the core biomedical databases.

### 2.2 Inclusion criteria

Studies were included if they met the following PICOS criteria: patients were adults with CT- or MRI-confirmed stroke and dysphagia documented by clinical assessment or instrumental examination (e.g., video fluoroscopic swallowing study or fiberoptic endoscopic evaluation), regardless of stroke type (ischemic/hemorrhagic), phase (acute/subacute/chronic), or geographic region. The intervention had to be video game-based with clearly defined game mechanics. The comparison could be conventional swallowing rehabilitation (e.g., oral motor exercises, swallowing maneuvers, verbal feedback training, or neuromuscular electrical stimulation). The outcome indicators are the Functional Oral Intake Scale (FOIS), the Penetration-Aspiration Scale (PAS), the Gugging Swallowing Screen (GUSS), the Dysphagia Outcome and Severity Scale (DOSS), Swallowing Quality of Life Questionnaire (SWAL-QOL), Dysphagia Handicap Index (DHI) and the Video Fluoroscopic Dysphagia Scale (VDS), among others, which are all indicators used to assess swallowing function. In addition, the type of study should be the randomized controlled trial (RCT).

### 2.3 Exclusion criteria

Studies were excluded if they met any of the following criteria: dysphagia caused by non-stroke etiologies (e.g., head and neck cancer, neurodegenerative diseases, or postsurgical conditions); interventions without explicit video game elements; review articles, conference abstracts, or animal studies; duplicate publications; or articles with unavailable full text or incomplete data.

### 2.4 Study selection and data extraction

Two reviewers independently performed the study selection. Duplicate records were first removed using EndNote reference management software. The reviewers then screened the titles and abstracts to exclude clearly irrelevant studies. Full texts of potentially eligible studies were retrieved and assessed against the inclusion criteria for final selection. Disagreements during the selection process were resolved through discussion or by consulting a third reviewer. The selection process was documented using a PRISMA flow diagram. Data extraction was also conducted independently by two reviewers using a pre-designed Excel form, which captured the following information: general study characteristics, patient characteristics, intervention details, comparison details, and outcome measures. Any disagreements during data extraction were resolved through discussion or by a third reviewer.

### 2.5 Risk of bias assessment

Two reviewers independently assessed the risk of bias in the included randomized controlled trials using Review Manager (RevMan) software across seven domains: random sequence generation, allocation concealment, blinding of participants and personnel, blinding of outcome assessment, incomplete outcome data, selective reporting, and other bias. Each domain was judged as low, high, or unclear risk based on reported information, with other potential biases (e.g., baseline imbalance, conflicts of interest) also evaluated. A risk of bias summary and graph were generated to visually present the bias profile of each study. Disagreements were resolved through discussion or by consulting a third reviewer. Additionally, the same two reviewers assessed the overall certainty of evidence for each outcome using the Grading of Recommendations Assessment, Development and Evaluation (GRADE) framework, rating evidence as high, moderate, low, or very low based on five domains: risk of bias, inconsistency, indirectness, imprecision, and publication bias. The GRADE assessments were summarized in a table and integrated into the interpretation of the findings.

### 2.6 Statistical analysis

Meta-analysis was performed using RevMan 5.4 software. For continuous outcomes, the weighted mean difference (WMD) with 95% confidence interval (CI) was calculated. For dichotomous outcomes, the odds ratio (OR) with 95% CI was calculated. Heterogeneity across studies was assessed using the *Q* test and *I*^2^ statistic. Given the anticipated variability among studies, a random-effects model was used for all analyses. An *I*^2^ value of 75% or greater indicated high heterogeneity, which warrants exploratory *post hoc* subgroup or sensitivity analyses to identify potential sources of heterogeneity. Sensitivity analysis was conducted by sequentially removing one study at a time to examine the robustness of the pooled effect estimates. If the removal of a particular study substantially changed the effect estimate (i.e., altered its direction or statistical significance), the impact of that study on the overall conclusion was evaluated and reported in the results. When ten or more studies were included, publication bias was assessed using funnel plots combined with Egger's regression test (26). Asymmetry in the funnel plot or a significant Egger's test (*P* < 0.05) was considered suggestive of publication bias.

## 3 Results

### 3.1 Study selection results

**Figure 1:**
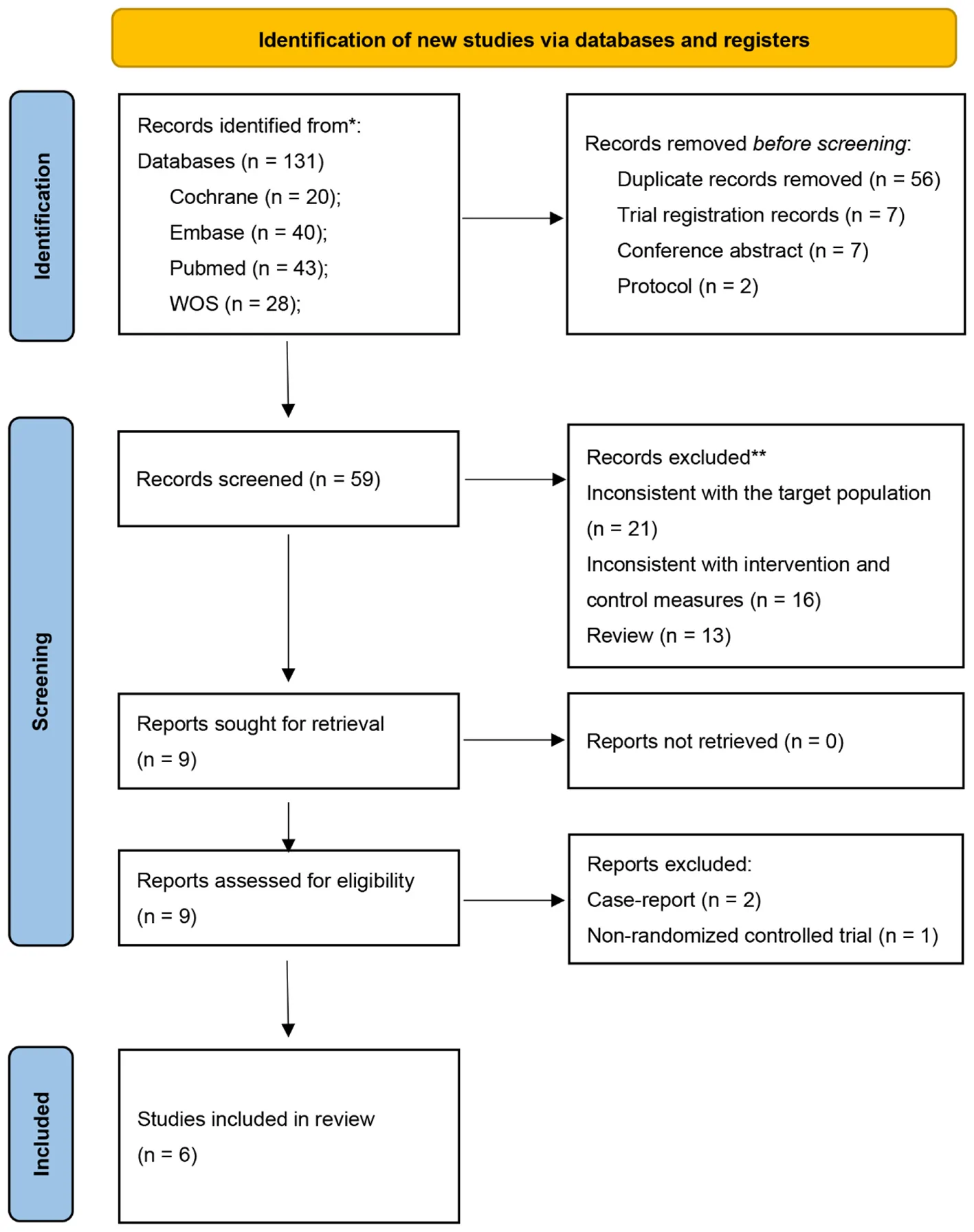
PRISMA flow diagram of the study selection process.

A systematic search of the four electronic databases (PubMed, Web of Science, Cochrane Library, and Embase) yielded 131 records: 43 from PubMed, 28 from Web of Science, 20 from Cochrane Library, and 40 from Embase. After importing all records into EndNote, 56 duplicate records were removed. Based on the document type classification in EndNote, 7 trial records, 7 conference abstracts, and 2 study protocol were excluded. Following screening of titles and abstracts, 50 records that clearly did not meet the inclusion criteria were excluded, with reasons as follows: the study population was not stroke patients with dysphagia (*n* = 21), the intervention did not include video game elements (*n* = 16), or the article was a review (*n* = 13). The full texts of the remaining 9 records were retrieved for detailed assessment. After full-text reading, two case reports and one non-randomized controlled study were further excluded. Finally, six studies were included in the systematic review and meta-analysis (Figure 1).

### 3.2 Basic characteristics of the included studies

Six studies published between 2019 and 2026 were included in this review, with a total sample size of 309 participants (151 in the intervention group and 158 in the control group), of whom 300 contributed data to the meta-analysis. The studies were conducted in Turkey, South Korea, and China (four studies). The majority of enrolled patients had ischemic stroke. All studies set cognitive inclusion criteria, requiring a Mini-Mental State Examination score of 22–27 or higher. The interventions in all included studies were video game-based training, although the core technologies differed across studies. The control groups in all studies received conventional swallowing rehabilitation. All the studies were randomized controlled trials, with training duration in the control groups matched to that of the intervention groups. The basic characteristics of the included studies are summarized in Table 1, with more detailed intervention details in Supplementary material 2.

**Table 1:** Characteristics of included studies.

Patient characteristics								Intervention group						
Study ID	Country/region	Study design	Sample size (total/ intervention/ control)	Stroke type	Disease duration (months)	Cognitive requirement	Game name	Core technology	Target muscles/actions	Intervention duration	Control group	Outcome measures	Assessment time points	Adverse events
Alyanak et al. (30)	Turkey	RCT	33 / 16 / 17	90.9% ischemic	Median 5 months	MMSE ≥24	Rose game, Bunny game	sEMG biofeedback	Suprahyoid muscles	15 sessions, 30 min/session, 3 weeks	Verbal feedback + conventional training	FOIS, PAS, DOSS, DHI	Baseline, post-intervention	Not reported
Hou et al. (32)	China	RCT	60/30/30	Mixed	< 0.5	MMSE >24	rabbit mountain-climbing game	sEMG biofeedback	Submental muscles	20 min/session, once daily, 7–14 days	direct feeding training + indirect sensory/motor training + tDCS	water swallowing test, FOIS, submental sEMG peak value, swallowing time limit, tongue pressure	Baseline, post-intervention	Not reported
Kang et al. (29)	China	RCT	60/30/30	Mixed	≈1	MMSE >27	“Mukbang” eating contest scenario	sEMG-triggered immersive virtual reality	Submental, hyoid muscles	30 min CST + VR training per session, 5 times/week for 3 weeks (15 sessions total)	Conventional swallowing therapy	WST, SSA, FOIS, sEMG root mean square (RMS) values	Baseline (pre-treatment) and after 3 weeks of treatment (post-treatment)	No serious training-related adverse events
Park et al. (28)	South Korea	RCT	46 (randomized), 37 (analyzed)/19/18	Mixed	3.6 ± 1.2	MMSE >22	LES 100 game system	Pressure sensor	Suprahyoid muscles	4 weeks, 5 × /week, 30 min/session	Traditional head-lift exercise	VDS, PAS, FOIS, compliance score	Baseline, 4 weeks	4 dropouts in control group due to neck pain
Zhang et al. (31)	China	RCT	26/14/12	78.6% ischemic	1.91 ± 0.85	MMSE ≥24	Carrot collecting, Maze navigation, Flying bird	Facial recognition (non-AI)	Labial, lingual, mandibular muscles	4 weeks, 5 × /week, 30 min/session	Traditional labial-lingual-mandibular training	GUSS, SSA, VVST, FOIS, SWAL-QOL, adherence	Baseline, 4 weeks	Not reported
Zhang et al. (27)	China	RCT	84/42/42	82.1% ischemic	1.72 ± 1.50	MMSE ≥24	Carrot collecting, Maze navigation, Flying bird	AI facial recognition (MediaPipe)	Labial, lingual, mandibular muscles	4 weeks, 5 × /week, 30 min/session	Traditional labial-lingual-mandibular training	GUSS, SSA, FOIS, MNA-SF, SWAL-QOL, adherence	Baseline, 4 weeks, 1-month follow-up	1 case of dizziness (self-resolved)

AI, artificial intelligence; CTAR, chin tuck against resistance; DHI, dysphagia handicap index; DOSS, dysphagia outcome and severity scale; EMG, electromyography; FOIS, functional oral intake scale; GUSS, gugging swallowing screen; MediaPipe, google's open-source multimedia machine learning framework; MMSE, mini-mental state examination; MNA-SF, mini-nutritional assessment short form; PAS, penetration-aspiration scale; RCT, randomized controlled trial; sEMG, surface electromyography; SSA, standardized swallowing assessment; SWAL-QOL, swallowing quality of life questionnaire; VDS, videofluoroscopic dysphagia scale; VFSS, videofluoroscopic swallowing study; VVST, volume-viscosity swallow test.

### 3.3 Risk of bias assessment results

**Figure 2:**
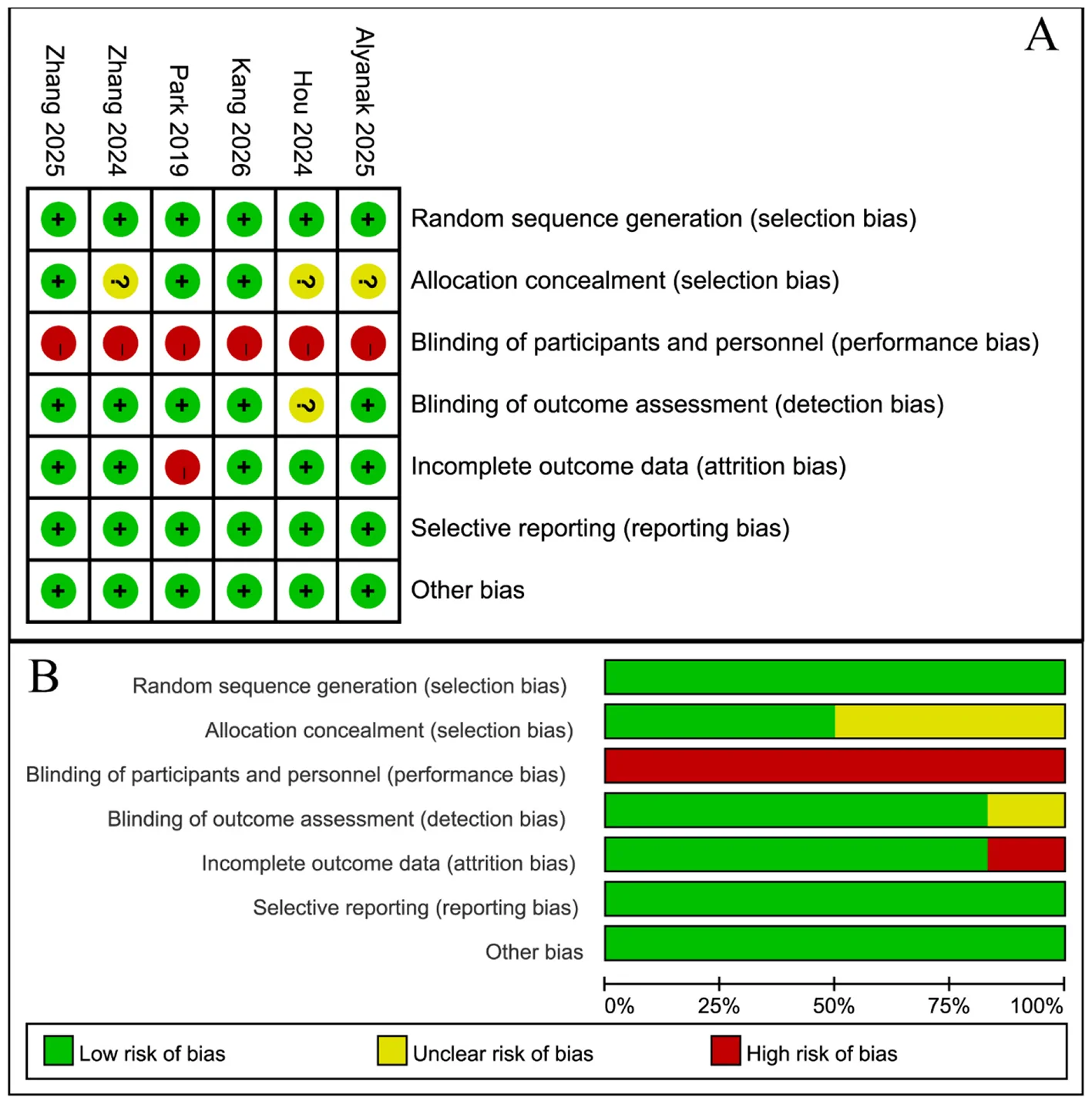
Risk of bias assessment. **(A)** Risk of bias summary; **(B)** Risk of bias graph.

All six studies reported the method of random sequence generation, and thus were rated as low risk in this field. Three studies (27–29) clearly reported the use of sealed, opaque envelopes for allocation concealment and was rated as low risk. The other studies (30–32) did not describe their allocation concealment methods and were rated as unclear risk. Due to the nature of the intervention (obvious differences between video game-based training and conventional rehabilitation), blinding of participants and personnel was not feasible in any of the six studies; all were rated as high risk for this domain. Hou et al. (32) did not report whether outcome assessors were blinded and was therefore rated as unclear risk for this domain. For incomplete outcome data, Park et al. (28) had nine dropouts and did not perform an intention-to-treat (ITT) analysis; only participants who completed the intervention were analyzed, which may have introduced bias. Thus, this study was rated as high risk. The risk of bias assessment results were presented in Figure 2.

### 3.4 Meta-analysis results

#### 3.4.1 Primary outcome

**Figure 3:**
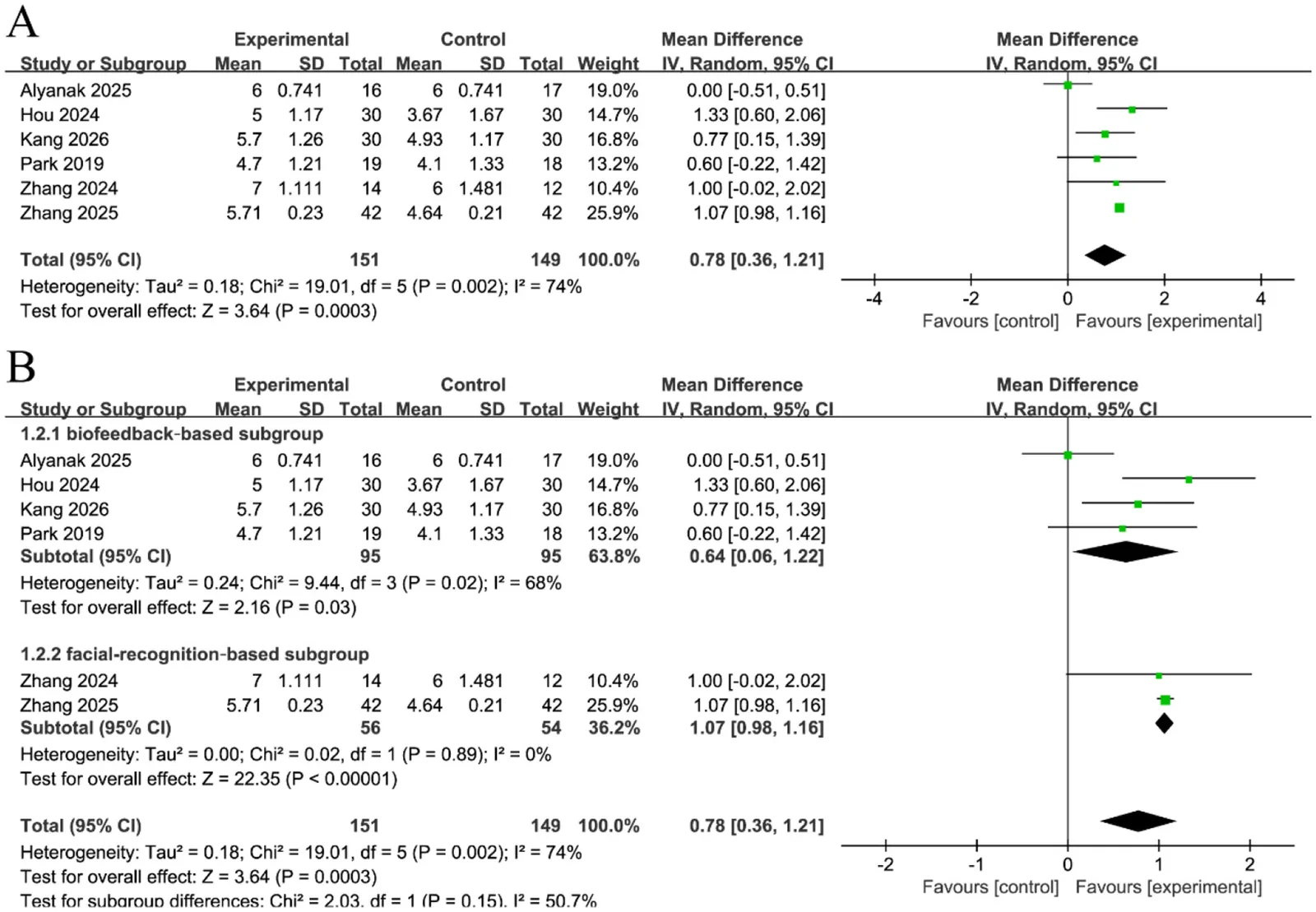
Forest plots of primary outcome. **(A)** Forest plots of the FOIS; **(B)** Forest plot of the subgroup analysis of FOIS.

A total of six studies reported FOIS scores, comprising 300 patients (151 in the intervention group and 149 in the control group). The pooled results from the random-effects model showed that video game-based interventions yielded a statistically significant improvement in FOIS scores compared with the control group (WMD = 0.78, 95% CI [0.36, 1.21], *P* = 0.0003); (Figure 3A). However, substantial heterogeneity was detected across the included studies (*I*^2^ = 74%, *P* = 0.002), indicating considerable between-study variation in treatment effect. To explore the potential sources of this heterogeneity, we performed an exploratory *post hoc* subgroup analysis stratified by the core technology of the video game interventions, categorizing them into biofeedback-based and facial-recognition-based subgroups (Figure 3B).

In the biofeedback-based subgroup (four studies, *n* = 190), the pooled estimate revealed a statistically significant improvement in FOIS scores between the intervention and control groups (WMD = 0.64, 95% CI [0.06, 1.22], *P* = 0.03), with substantial heterogeneity (*I*^2^ = 68%). In the facial-recognition-based subgroup (two studies, *n* = 110), the video game intervention also demonstrated a statistically significant improvement in FOIS scores compared with the control group (WMD = 1.07, 95% CI [0.98, 1.16], *P* < 0.00001), with no evidence of heterogeneity across studies (*I*^2^ = 0%). The test for subgroup differences showed a trend toward between-group difference, but it was not statistically significant (Chi^2^ = 2.03, *P* = 0.15, *I*^2^ = 50.7%). Given the *post hoc* nature of this analysis and the small number of studies per subgroup, these results were considered hypothesis-generating rather than confirmatory.

Sensitivity analysis for FOIS revealed that the overall effect was sensitive to the inclusion of Alyanak et al. (30); excluding this study substantially reduced statistical heterogeneity while preserving the direction of the pooled effect (WMD = 1.06, 95% CI [0.97, 1.15], *P* < 0.00001; *I*^2^ = 0%); (Supplementary material 3). Within the biofeedback-based subgroup, excluding Alyanak et al. (30) also reduced heterogeneity substantially (WMD = 0.90, 95% CI [0.49, 1.31], *P* < 0.0001; *I*^2^ = 1%), whereas the facial-recognition-based subgroup remained unchanged. The results of the difference analysis between the two groups are more stable (Ch*I*^2^ = 0.60, *P* = 0.44, *I*^2^ = 0%). This restricted analysis was considered exploratory and did not replace the primary analysis that included all eligible studies. Excluding any other single study did not substantially alter the direction or significance of the overall effect.

#### 3.4.2 Secondary outcomes

For secondary outcomes, quantitative meta-analysis was performed only for those reported by at least two studies. Accordingly, the SSA and swallowing-related quality of life measures (SWAL-QOL) met this threshold and were pooled.

##### 3.4.2.1 Standardized swallowing assessment (SSA)

**Figure 4:**
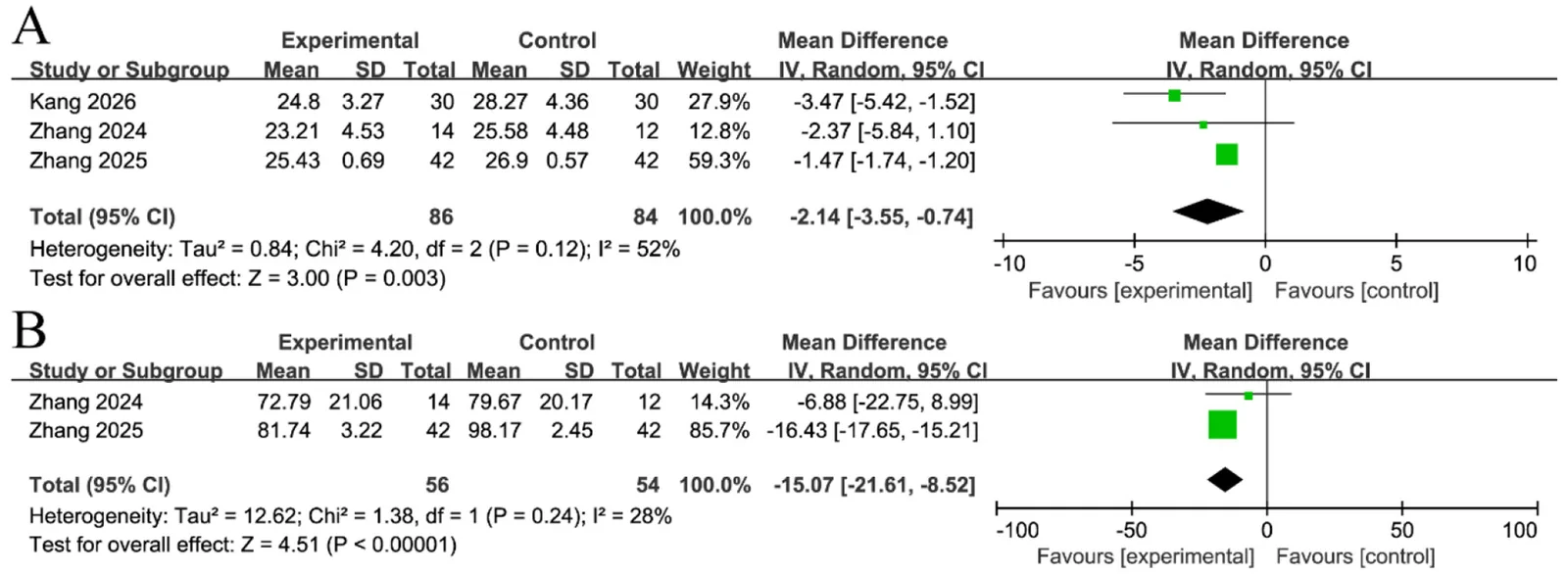
Forest plots of secondary outcomes. **(A)** Forest plot of the SSA; **(B)** Forest plot of the SWAL-QOL.

Three studies involving 170 patients (86 in the intervention group and 84 in the control group) reported the SSA score. The pooled results showed that the intervention group had significantly lower SSA scores than the control group (WMD = −2.14, 95% CI [−3.55, −0.74], *P* = 0.003); (Figure 4A), indicating that video game-based interventions significantly improved swallowing function (lower SSA scores indicate better swallowing function). Moderate heterogeneity was detected (*I*^2^ = 52%, *P* = 0.12).

##### 3.4.2.2 Swallowing-related quality of life

Two studies involving 110 patients reported SWAL-QOL. In these two included studies, lower SWAL-QOL scores indicated better swallowing-related quality of life; as both studies adopted the same scoring direction, no further transformation was applied before pooling. The pooled results showed that the intervention group had significantly lower (better) scores than the control group (WMD = −15.07, 95% CI [−21.61, −8.52], *P* < 0.00001); (Figure 4B), indicating that video game-based interventions significantly improved swallowing-related quality of life. No substantial heterogeneity was detected (*I*^2^ = 28%, *P* = 0.24). In addition, Alyanak et al. (30) reported similar results using the DHI scale, the intervention group showed significantly lower DHI scores (41 [32–50] vs. 60 [45–73], *P* = 0.041) and a greater median reduction from baseline (−12 [−21.5 to −6] vs. −4 [−6 to −2], *P* < 0.001), indicating improved swallowing-related quality of life.

### 3.5 GRADE evidence quality assessment

Based on the GRADE framework, the quality of evidence for the primary outcome FOIS was rated as low when all six studies were pooled (WMD = 0.78, 95% CI [0.36, 1.21]), with downgrading due to serious risk of bias and serious inconsistency (*I*^2^ = 74%). However, after excluding Alyanak et al. (30), the heterogeneity resolved completely, and the evidence quality improved to moderate (WMD = 1.06, 95% CI [0.97, 1.15]), with downgrading only for risk of bias. The secondary outcome SSA provided moderate quality evidence, while the SWAL-QOL outcome was rated as low quality, mainly because of serious imprecision related to the small sample size and wide confidence interval. No downgrading was applied for indirectness or publication bias for any of the pooled outcomes (Supplementary material 4).

## 4 Discussion

This systematic review and meta-analysis of six RCTs (*n* = 309) demonstrates that video game-based interventions significantly improve swallowing function in patients with post-stroke dysphagia (FOIS: WMD = 0.78, 95% CI [0.36, 1.21], *P* = 0.0003). However, substantial heterogeneity (*I*^2^ = 74%) lowered the GRADE certainty of this overall estimate to low. Sensitivity analysis identified Alyanak et al. (30) as the primary source of heterogeneity, excluding this study reduced *I*^2^ to 0% while maintaining the direction of the pooled effect, raising the GRADE certainty to moderate in this exploratory restricted analysis (WMD = 1.06, 95% CI [0.97, 1.15]). However, this restricted analysis does not replace the primary analysis that included all eligible studies. Notably, Alyanak et al. (30) reported outcomes as medians and interquartile ranges. We converted these into means and standard deviations using a tool developed by Chinese scholars (33), which may have contributed to the observed heterogeneity. An exploratory *post hoc* subgroup analysis revealed that facial-recognition-based interventions (two studies, *n* = 110) produced larger and more consistent FOIS improvements (WMD = 1.07, 95% CI [0.98, 1.16], *I*^2^ = 0%) than biofeedback-based interventions (four studies, *n* = 190; WMD = 0.64, 95% CI [0.06, 1.22], *I*^2^ = 68%). However, excluding Alyanak et al. (30) from the biofeedback subgroup substantially reduced heterogeneity (*I*^2^ from 68 to 1%), indicating that the inconsistency within this subgroup was also largely attributable to this single study. Video game-based interventions also significantly improved SSA scores (WMD = −2.14, 95% CI [−3.55, −0.74]; moderate GRADE certainty) and swallowing-related quality of life (WMD = −15.07, 95% CI [−21.61, −8.52]; low GRADE certainty due to imprecision). Taken together, these findings support video game-based rehabilitation as a promising adjunctive approach.

Video game-based interventions may improve swallowing function through general mechanisms rooted in motor learning and neuroplasticity. Based on motor learning theory, external feedback (visual and auditory) accelerates motor skill acquisition (18, 19). Video games provide real-time, dynamic audiovisual feedback, allowing patients to adjust their swallowing movements immediately and thereby improve training precision. This stands in sharp contrast to the delayed verbal feedback often used in conventional rehabilitation (32). Besides, through the cultivation of flow experiences and grit, gamified design significantly increases patients' treatment motivation and adherence (23), enabling them to complete more intensive and sustained training (34). Neuroplasticity is a core mechanism of functional recovery after stroke (35). Evidence indicates that video games can induce neuroplasticity, thereby promoting cortical reorganization, motor recovery, cognitive improvement, and the transfer of spatial knowledge to daily living environments (36). In addition, the multisensory stimulation provided by video games may facilitate the reconstruction and optimization of the swallowing reflex arc by enhancing sensory input (37).

In an exploratory *post hoc* subgroup analysis, the subgroup difference did not reach statistical significance. The numerically larger point estimate and consistently zero heterogeneity observed with facial-recognition-based systems warrant exploratory consideration, but these findings remain strictly hypothesis-generating and should not be interpreted as evidence of superiority of one technology over another. Facial-recognition-based systems simultaneously train multiple orofacial muscle groups (labial, lingual, and mandibular) through comprehensive facial movements such as cheek puffing, tongue protrusion, and chin pressing, better approximating the coordinated nature of physiological swallowing (38). In contrast, biofeedback-based systems typically focus on a single muscle group, most commonly the suprahyoid muscles via sEMG. While targeted suprahyoid training is important for laryngeal elevation and airway protection, it may be insufficient for patients with impairments affecting multiple phases of swallowing (39). Additionally, facial-recognition systems provide real-time, objective feedback on movement accuracy and completeness (40), whereas biofeedback systems often rely on threshold-based binary feedback (e.g., reaching a target pressure or EMG amplitude). The richer, more discriminative feedback from facial-recognition systems may facilitate more precise motor learning (41). The visual engagement of facial movement-controlled games may also enhance patients' awareness of orofacial movements, potentially promoting cortical reorganization through increased sensorimotor integration (42). Nevertheless, given the non-significant subgroup difference and the small number of studies within each subgroup, these mechanistic interpretations remain speculative and should be viewed as hypothesis-generating rather than confirmatory.

Video game-based interventions hold substantial potential for home-based application, which could help address the shortage of professional rehabilitation resources, especially in community-based or remote rehabilitation settings (20). The findings of this study offer several practical implications for clinical practice. The moderate-certainty evidence supporting FOIS improvement (after excluding the outlying study) and SSA reduction suggests that video game-based training can be a useful adjunct to conventional rehabilitation, particularly when improving patient adherence is a priority. For patients requiring enhanced laryngeal elevation, biofeedback-based games (e.g., EMG-driven or pressure-based systems) may be appropriate (30, 43); for those needing comprehensive training of the lips, tongue, and jaw, facial-recognition-based games may offer additional advantages with more consistent evidence (27, 31). However, the low-certainty evidence for the overall FOIS estimate and for quality-of-life outcomes indicates that these interventions should not replace conventional therapy. Shared decision-making with patients is essential, acknowledging the current limitations in the evidence base.

Several limitations warrant consideration. All included studies had high risk of performance bias due to the inability to blind participants and personnel to the intervention; some studies did not adequately describe allocation concealment. The overall FOIS estimate was downgraded to low certainty due to substantial heterogeneity (*I*^2^ = 74%), and while sensitivity analysis identified Alyanak et al. (30) as the primary source, the presence of this outlier introduces uncertainty. The SWAL-QOL analysis was downgraded to low certainty due to imprecision (small sample size, wide confidence interval). Additionally, the small number of studies precluded formal publication bias assessment, and other potential sources of heterogeneity (e.g., disease phase, dysphagia severity) could not be explored. In the pooled analyses of secondary outcomes, the study by Zhang et al. (27) carried a disproportionately large weight (59.3% for SSA and 85.7% for SWAL-QOL), indicating that these summary estimates are largely driven by a single trial. This overweighting undermines the robustness of the secondary findings and highlights the urgent need for more large-scale, multicenter RCTs to balance the evidence base and validate these results. Sensitivity analyses excluding this study are warranted but were precluded by the limited number of included trials. Future large-sample, multicenter RCTs with rigorous methodological design are urgently needed. Head-to-head trials directly comparing facial-recognition-based and biofeedback-based systems are particularly important to validate the subgroup findings. Neuroimaging studies (e.g., fNIRS, fMRI) could elucidate the neuroplasticity mechanisms underlying different types of game interventions.

## 5 Conclusion

This meta-analysis of six RCTs provides low- to moderate-certainty evidence suggesting that video game-based interventions may improve swallowing function and quality of life in patients with post-stroke dysphagia. The overall FOIS estimate carries low GRADE certainty due to substantial heterogeneity, but sensitivity analysis excluding an outlying study yields moderate-certainty evidence (WMD = 1.06, 95% CI [0.97, 1.15]; *I*^2^ = 0%). The exploratory *post hoc* subgroup analysis suggested that both biofeedback-based and facial-recognition-based technologies are effective, with the latter showing numerically larger and more consistent effects; however, the subgroup difference was not statistically significant, and these findings remain exploratory. Given the limited number of trials, high risk of performance bias, and variable GRADE certainty across outcomes, these results should be interpreted cautiously. Large-scale, methodologically rigorous RCTs with long-term follow-up are urgently needed to confirm clinical utility and establish optimal training protocols.
