# Supplementary material for: Video game-based interventions for post-stroke dysphagia: a systematic review and meta-analysis
Source: Front Neurol. 2026 Aug 28;17:1889031. doi: 10.3389/fneur.2026.1889031 (PMC13563507; doi:10.3389/fneur.2026.1889031)
Supplement: Supplementary file 1 [file Table_3.docx]

Figure S1. Forest plots and subgroup analysis of FOIS scores after sensitivity analysis (excluding Alyanak 2025).

(A) Overall pooled analysis of the remaining 5 studies (Heterogeneity: I²=0%); (B) Subgroup analysis based on intervention type (biofeedback-based subgroup vs. facial-recognition-based subgroup).

| Omitted Study | Effect Size (WMD) | 95% Confidence Interval | Heterogeneity (I²) |
| --- | --- | --- | --- |
| **None (All included)** | **0.78** | **[0.36, 1.21]** | **74%** |
| **Exclude Alyanak 2025** | 1.06 | [0.97, 1.15] | 0% |
| **Exclude Hou 2024** | 0.69 | [0.19, 1.18] | 78% |
| **Exclude Kang 2026** | 0.79 | [0.27, 1.30] | 78% |
| **Exclude Park 2019** | 0.81 | [0.34, 1.29] | 78% |
| **Exclude Zhang 2024** | 0.76 | [0.28, 1.23] | 79% |
| **Exclude Zhang 2025** | 0.69 | [0.18, 1.19] | 60% |

Table S1. Sensitivity analysis (leave-one-out) table for the FOIS outcome

Figure S2 Overall forest plot of FOIS with all studies included (Primary analysis)

Figure S3. Forest plot of FOIS after excluding Alyanak 2025 (Sensitivity analysis)

Figure S4. Forest plot of FOIS after excluding Hou 2024 (Sensitivity analysis)

Figure S5. Forest plot of FOIS after excluding Kang 2026 (Sensitivity analysis)

Figure S6. Forest plot of FOIS after excluding Park 2019 (Sensitivity analysis)

Figure S7. Forest plot of FOIS after excluding Zhang 2024 (Sensitivity analysis)

Figure S8. Forest plot of FOIS after excluding Zhang 2025 (Sensitivity analysis)
