# Supplementary material for: Video game-based interventions for post-stroke dysphagia: a systematic review and meta-analysis
Source: Front Neurol. 2026 Aug 28;17:1889031. doi: 10.3389/fneur.2026.1889031 (PMC13563507; doi:10.3389/fneur.2026.1889031)
Supplement: Supplementary file 2 [file Table_4.docx]

**GRADE evidence quality assessment for primary and secondary outcomes**

| Outcome | Study design | Risk of bias | Inconsistency | Indirectness | Imprecision | Publication bias | Effect estimate (WMD, 95% CI) | Quality of evidence | Importance |
| --- | --- | --- | --- | --- | --- | --- | --- | --- | --- |
| **FOIS** (before excluding Alyanak 2025) | RCT (6 studies, 300 patients) | **Serious**① | **Serious**⑦ | **Not downgraded** | **Not downgraded** | **Not downgraded**③ | 0.78 [0.36, 1.21] | **⊕⊕◯◯ Low** | Critical |
| **FOIS** (after excluding Alyanak 2025) | RCT (5 studies, 267 patients) | **Serious**① | **Not downgraded**② | **Not downgraded** | **Not downgraded** | **Not downgraded**③ | 1.06 [0.97, 1.15] | **⊕⊕⊕◯ Moderate** | Critical |
| **SSA** | RCT (3 studies, 170 patients) | **Serious**① | **Not downgraded**④ | **Not downgraded** | **Not downgraded** | **Not downgraded**③ | –2.14 [–3.55, –0.74] | **⊕⊕⊕◯ Moderate** | Important |
| **SWAL-QOL** (reverse-scored) | RCT (2 studies, 110 patients) | **Serious**① | **Not downgraded**⑤ | **Not downgraded** | **Serious**⑥ | **Not downgraded**③ | –15.07 [–21.61, –8.52] | **⊕⊕◯◯ Low** | Important |

Detailed rationale for downgrading:

① Risk of bias (downgraded by one level): All included studies were rated as having a high risk of performance bias, as blinding of participants and personnel was not feasible due to the nature of the interventions (video game‑based training vs. conventional rehabilitation). Some studies did not adequately describe allocation concealment methods and were rated as unclear risk. In addition, Park et al. (2019) had a high dropout rate and did not perform an intention‑to‑treat (ITT) analysis, introducing potential attrition bias. Taken together, these limitations warranted downgrading by one level.

② Inconsistency for FOIS (after excluding Alyanak 2025): After excluding Alyanak 2025—which was identified as the major source of heterogeneity—the sensitivity analysis showed no evidence of heterogeneity across the remaining studies (I² = 0%, P = 0.62). Therefore, no downgrading was applied.

③ Publication bias: As fewer than 10 studies were available for each outcome, formal tests (e.g., funnel plot or Egger's test) could not be reliably performed. No downgrading was applied.

④ Inconsistency for SSA: The heterogeneity test showed I² = 52%, P = 0.12, indicating moderate but statistically non‑significant heterogeneity. Given the small number of included studies, no downgrading was applied.

⑤ Inconsistency for SWAL-QOL: The heterogeneity test showed I² = 28%, P = 0.24, indicating low heterogeneity. No downgrading was applied.

⑥ Imprecision for SWAL-QOL (downgraded by one level): The total sample size was only 110 patients, and the 95% confidence interval was relatively wide (range: 13.09 points). Although the interval did not cross the null effect, the precision of the effect estimate was suboptimal, warranting downgrading by one level.

⑦ Inconsistency for FOIS (before excluding Alyanak 2025) – downgraded by one level: The heterogeneity test across all six studies showed substantial statistical heterogeneity (I² = 74%, P = 0.002). Sensitivity analysis identified Alyanak 2025 as the primary source of this variation. Given the considerable unexplained heterogeneity in the pooled estimate, downgrading by one level was applied.
